# Golgi clustering by the deficiency of COPI-SNARE in *Drosophila* photoreceptors

**DOI:** 10.3389/fcell.2024.1442198

**Published:** 2024-09-04

**Authors:** Tatsuya Tago, Yumi Yamada, Yumi Goto, Kiminori Toyooka, Yuka Ochi, Takunori Satoh, Akiko K. Satoh

**Affiliations:** ^1^ Program of Life and environmental Science, Graduate School of Integral Science for Life, Hiroshima University, Hiroshima, Japan; ^2^ Technology Platform Division, Mass Spectrometry and Microscopy Unit, RIKEN Center for Sustainable Resource Science, Yokohama, Japan

**Keywords:** *Drosophila*, photoreceptors, recycling endosomes, Golgi stacks, BFA-body

## Abstract

A comprehensive study of soluble *N*-ethylmaleimide-sensitive factor attachment protein receptors (SNAREs) in the fly genome by RNAi in *Drosophila* photoreceptors indicated that knockdown of any of the COPI-SNAREs, *Syx18*, *Sec20*, and *Use1*, resulted in the same characteristic phenotypes: Golgi stacks gathering on their *trans*-side, laterally expanded Golgi cisternae, and a reduced number of discrete Golgi stacks. These Golgi stacks are reminiscent of mammalian Golgi ribbons and Brefeldin A (BFA)-bodies in *Drosophila* S2 cells. As previously reported, BFA suppresses *trans*-Golgi network (TGN) fission and Golgi stack separation to form a BFA-body, which is a cluster of Golgi stacks cored by recycling endosomes. We found that the impairing each of COPI-SNAREs results in clustered Golgi stacks similar to BFA-bodies, indicating that COPI-SNAREs have a role to separate clustered Golgi stacks. These results further support the idea that the movement of Golgi stacks and the balance of fusion and fission of the TGN determine the level of clustering and ribbon formation of Golgi stacks within cells.

## 1 Introduction

The basic units of the Golgi apparatus are the Golgi stacks, which are composed of several flattened membrane structures called cisternae and membrane networks on both sides (Nakano, 2022; Papanikou and Glick, 2014). The organization of Golgi stacks within cells differs between species. In plants and many invertebrates including *Drosophila* and *C. elegans*, dozens of Golgi stacks are separated and scattered throughout the cytoplasm (Ito and Uemura, 2022; Kondylis and Rabouille, 2009). However, in mammalian and other animal cells, Golgi stacks gather in the perinuclear area near the centrosome and form a gigantic Golgi ribbon (Benvenuto et al., 2024; Klumperman, 2011; Saraste and Prydz, 2019; Terasaki, 2000). The formation of Golgi ribbons depends on the accumulation of Golgi stacks near the centrosome via dynein-dependent movement along microtubules (Yadav and Linstedt, 2011; Yadav et al., 2012). Hundreds of scattered Golgi stacks were observed in microtubule-depolymerized cells, similar to those observed in invertebrates or plant cells. The function and mechanism of Golgi ribbon formation are not clear, but this Golgi configuration seems to be important for cellular physiology. Golgi ribbons are disassembled and reassembled during cell division under normal conditions and fragmented under pathological conditions, including neurodegeneration and cancer (Caracci et al., 2019; Gosavi and Gleeson, 2017; Marie et al., 2012; Saraste and Prydz, 2019).

Soluble *N*-ethylmaleimide-sensitive factor attachment protein receptors (SNAREs) are a family of small conserved eukaryotic proteins responsible for most intracellular fusion events of organellar trafficking (Grissom et al., 2020; Hong and Lev, 2014; Jahn et al., 2024; Stanton and Hughson, 2023). SNAREs comprise approximately 38 members in humans and 25 members in *Drosophila*. SNAREs involved in transport between the ER and Golgi are well known in yeast and mammalian cells (Linders et al., 2019). *Syx5*, *Bos1* (“*Membrin*” in flies), *Bet1*, and *Sec22* are SNAREs for anterograde transport from ER to Golgi, which regulate the fusion of COPII vesicles to cis-Golgi cisternae. We refer to them as COPII-SNAREs. Syx18, Sec20, Use1, and Sec22 are SNAREs involved in the retrograde transport from the Golgi apparatus to the ER, which regulates the fusion of COPI vesicles to cis-Golgi cisternae. We refer to them as COPI-SNAREs (Grissom et al., 2020; Hong and Lev, 2014).

We performed RNAi screening of SNAREs using mosaic expression of RNAi constructs in *Drosophila* retinas and found that the knockdown phenotype of SNAREs involved in the transport between the ER and Golgi is characteristic of severe rhodopsin 1 (Rh1) reduction but no accumulation of Rh1 in the cytoplasm, suggesting degradation of Rh1 by ER-associated degradation (Ochi et al., unpublished). In this study, we investigated the effects of SNARE deficiency on Golgi morphology. We found that the deficiency of SNAREs involved in COPII fusion causes the transformation of Golgi stacks into vesicle clusters, whereas the deficiency of SNAREs involved in COPI fusion causes Golgi stacks to cluster together.

## 2 Materials and methods

### 2.1 *Drosophila* stocks and genetics

The flies were grown at 20°C–25°C on standard cornmeal–glucose–agar–yeast food. The following fly stocks were used: Rh1-Gal4 (Dr. Hama, Kyoto Sangyo University), UAS-CFP::GalT (Satoh et al., 2005), and UAS-Syx5::Myc (Dr. Burke, Monash University). Fly lines with RNAi constructs of SNAREs for the transport between ER and Golgi, which were used in this study, were *Syx5* ^*JF03330*^ (Bloomington *Drosophila* Stock Center, stock number 11678, Bloomington, IN, United States: BL11678), *Bet1* ^*HMJ22351*^ (BL58269), *Sec22* ^*HMS01238*^ (BL34893), *Use1* ^*GLC01442*^ (BL43253), *Sec20* ^*HMS01172*^ (BL34693), *Syx18* ^*KK101345*^ (Viena *Drosophila* Resource Center, stock number 105113, Viena, Austria: v105113) and *Membrin* ^GD2313^ (v44534). The *Syx18* ^KK101345^ RNAi construct was on the 30B landing site rather than on the 40D landing site (Green et al., 2014). To obtain SNARE knockdown mosaic retinas, we crossed them with CoinFLP-Act5C-Gal4 (BL58751) (Bosch et al., 2015) or CoinFLP-longGMR-Gal4 (Ochi et al., in co-submission).

### 2.2 Immunohistochemistry

Fixation and staining were performed as previously described (Otsuka et al., 2019; Satoh and Ready, 2005). Primary antisera were as follows: rabbit anti-Rh1 (1:1000) (Satoh et al., 2005), mouse monoclonal anti-Na^+^/K^+^-ATPase alpha subunit (*α5*: 1:500 ascite; Developmental Studies Hybridoma Bank (DSHB), Iowa City, IA, United States), rabbit anti-Myc (1:300) (Medical and Biological Laboratories, Nagoya, Japan; No. 562), rabbit anti-Sec22 (1:300) (Dr. Paden, University of Sheffield, United Kingdom), rabbit anti-GM130 (1:300) (Abcam #ab30637, Cambridge, United Kingdom), Goat anti-Golgin245 (1:300) (DSHB) (Riedel et al., 2016), Goat anti-GMAP (1:300) (DSHB) (Riedel et al., 2016), guinea pig anti-αCOP (1:150) (a gift from Dr. Inoue, Kyoto Sangyo University, Kyoto, Japan) (Kitazawa et al., 2012), rabbit anti-MPPE (1:1000) (a gift from Dr. Han, Southeast University, Nanjing, China), rat anti-Rab11 (1:300) (Otsuka et al., 2019) and Guinea pig anti-Rab6 (1:300) (Iwanami et al., 2016). The secondary antibodies used were anti-mouse, anti-goat, anti-rabbit, and anti-rat antibodies labelled with Alexa Fluor 488, 568, and 647 (1:300; Life Technologies, Carlsbad, CA, United States). Phalloidin-conjugated Alexa Fluor 568 (1:100; Life Technologies) was used for F-actin staining. Images of the samples were recorded using an FV3000 confocal microscope (UPLXAPO60XO 1.30 NA and UPlanSApo 60 × S2 1.42 NA objective lens; Olympus, Tokyo, Japan). To minimize bleed-through, each signal in double- or triple-stained samples was sequentially imaged. Images were processed in accordance with the Guidelines for Proper Digital Image Handling using ImageJ and/or Affinity Photo (Serif Europe Ltd., West Bridgford, Nottinghamshire, United Kingdom) (Schindelin et al., 2012). For the quantification of the mean gray value of Rh1, MPPE, αCOP, and GM130 staining in photoreceptor cells, we used more than 3 mosaic retinas. The areas of cytoplasm or whole cells and their staining intensities were measured using Fiji.

The projection images made from 9 slices of z-stacks with 0.4 μm were used for quantification of Golgi number and morphology. For quantification of Golgi number, GMAP staining was subjected to “analyze particles” in Fiji. Area is defined as the number of pixels of a single Golgi, which we obtained by “analyze particles.” Circularity (Circ) was calculated as 4π*area/perimeter^2. The aspect ratio (AR) was calculated for the major or minor axis. To quantify Golgi morphology, we measured 173 Golgi stacks in wild-type cells, 110 Golgi stacks in *Use1* RNAi photoreceptors in *Use1* RNAi mosaic retinas, 94 Golgi stacks in wild-type cells, and 268 Golgi stacks in *Syx5* RNAi photoreceptors in *Syx5* RNAi mosaic retinas.

### 2.3 Electron microscopy

Electron microscopy was performed as previously described (Satoh et al., 1997). The samples were observed under a JEM1400 electron microscope (JEOL, Tokyo, Japan), and montages were prepared using a CCD camera system (JEOL). The phenotypes were investigated using sections at the depth at which a couple of photoreceptor nuclei within the ommatidia were observed. The vesicle diameter was determined as previously described (Yang et al., 2021).

### 2.4 Serial section scanning electron microscopy observation of *Syx18* or *Bet1* RNAi-expressing photoreceptors

Serial-section scanning electron microscopy was performed using a high-resolution field-emission scanning electron microscope and a back-scattered electron detector. Serial ultrathin sections (thickness: 50 nm) were cut using a diamond knife (Diatome 45°) on an ultramicrotome (EM UC7, Leica Microsystems, Wetzlar, Germany) and placed on silicon wafers (10 × 22 mm). The sections were stained with 0.4% uranyl acetate for 10 min and lead stain solution (Sigma-Aldrich, St. Louis, MO, United States) for 2 min and coated with osmium tetroxide using an osmium coater (HPC-1SW, Vacuum Device Inc., Mito, Japan). Serial sections were observed at an accelerating voltage of 2 kV using a field-emission scanning electron microscope (Regulus8240; Hitachi High-Tech, Tokyo, Japan) equipped with an auto-capture for array tomography and a low-angle backscattered electron detector.

### 2.5 Ethics statement

This manuscript presents research on animals that does not require ethical approval for their study.

## 3 Results

### 3.1 Great reduction of Rh1 in the rhabdomere without cytoplasmic accumulation by COPI- and COPII-SNARE knockdown

**FIGURE 1:**
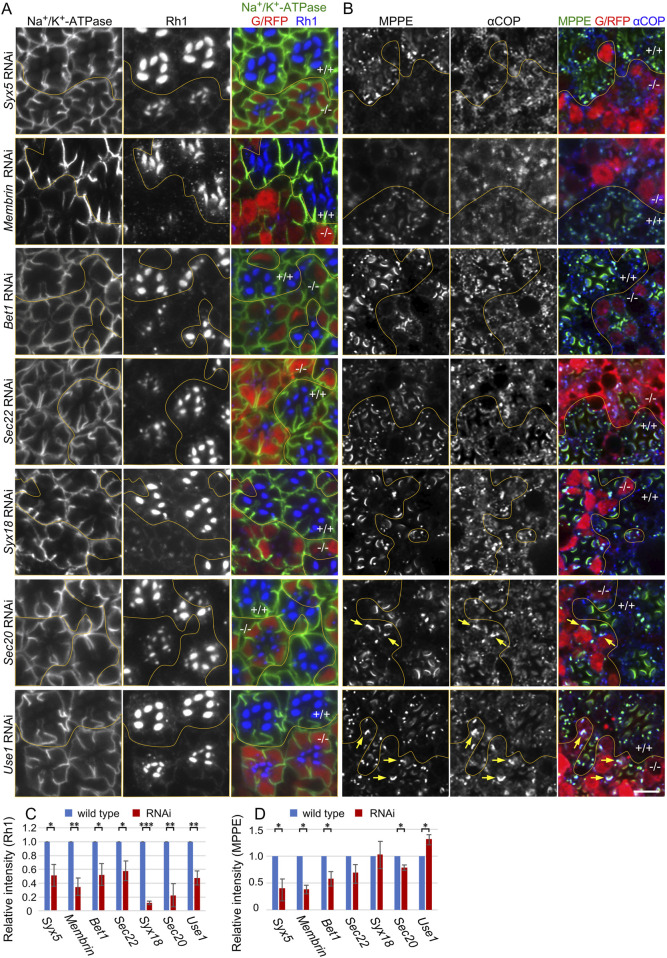
Reduction of Rh1 in the rhabdomeres by knockdown of SNAREs for COPI and COPII fusion. **(A)** Immunostaining of SNARE RNAi construct-expressing retina by eyeless-CoinFLP-longGMR-Gal4 (*Syx5*, *Bet1*, *Syx18*, and *Sec20*) or eyeless-CoinFLP-Act5C-Gal4 (*Membrin*, *Sec22*, and *Use1*) using anti-Na^+^/K^+^-ATPase-α (green) and anti-Rh1 (blue) antibodies. RFP/GFP (red) represents the cells expressing RNAi constructs. **(B)** Immunostaining of SNARE RNAi construct-expressing retina by eyeless-CoinFLP-longGMR-Gal4 (*Syx5*, *Bet1*, *Syx18*, and *Sec20*) or eyeless-CoinFLP-Act5C-Gal4 (*Membrin*, *Sec22*, and *Use1*) using anti-MPPE (green) and anti-αCOP (blue) antibodies. RFP/GFP (red) represents the cells expressing RNAi constructs. The anti-MPPE antibody stains medial Golgi and also rhabdomere tips, the latter is likely representing cross-reactivity. **(C, D)** The ratio of integrated fluorescence density for Rh1 **(C)**, and MPPE **(D)** staining of the cytoplasm compared to that of whole cells was plotted. Blue bars indicate wild-type cells and red bars indicate cells expressing RNAi constructs. Error bars indicate the SD of three retinas. Significance according to two-tailed unpaired Student’s t*-*test: **p < 0.01 and *p < 0.05. Scale bar: 5 μm **(A, B)**.

Through a comprehensive study of SNAREs in the fly genome following RNAi knockdown, we classified the phenotypes caused by the SNARE RNAi knockdown into three categories. Category III represents RNAi lines that induce a significant reduction in Rh1 in the rhabdomeres without any accumulation of Rh1 in the cytoplasm (Ochi et al., in co-submission). This phenotype of Category III RNAi lines was identical to that caused by the *Syx5* hypomorphic allele *Syx5* ^*EP2313*^ (Satoh et al., 2016). RNAi lines targeting SNAREs involved in COPII and COPI fusion (COPII- and COPI-SNAREs), *Membrin*, *Bet1*, *Sec22*, *Syx18*, *Sec20*, and *Use1* are all categorized as Category III (Ochi et al., in co-submission). Figure 1A shows the localization of Rh1 and Na^+^/K^+^ **-**ATPase in *Syx5*-, *Membrin-*, *Bet1*-, *Sec22*-, *Syx18*-, *Sec20*-, and *Use1* RNAi-expressing retinas. In all six retinas, SNARE-reduced photoreceptors showed a significant reduction in Rh1 but no accumulation of Rh1 in the cytoplasm, similar to the findings of a previous study (Ochi et al., in co-submission). Quantification of Rh1 signal intensities indicated 42%–88% reduction in Rh1 by SNARE RNAi expression (Figure 1C). In contrast, the reduction in the Na^+^/K^+^ **-**ATPase activity was limited (Figure 1A, green).

As knockdown of COPI- and COPII-SNAREs are expected to affect Golgi morphology and function, we next investigated the localization of metallophosphoesterase **(**MPPE), a transmembrane protein in *medial*-Golgi cisternae (Cao et al., 2011) and also αCOP, a subunit of COPI (Figure 1B). The amount of MPPE in the COPII-SNARE RNAi-expressing photoreceptors was greatly reduced. Quantification of MPPE signal intensities indicated a 30%–60% reduction in MPPE by COPII-SNARE RNAi (Figure 1D). However, the amount of MPPE in the COPI-SNARE RNAi-expressing photoreceptors remained unaffected (Figures 1B,D). As a transmembrane protein, MPPE is expected to behave as a cargo for COPI and COPII vesicles; these results could be interpreted as indicating that MPPE transport from ER to Golgi is inhibited in COPII-SNARE but not COPI-SNARE RNAi expression. Interestingly, the medial Golgi cisternae visualized by MPPE immunostaining appeared elongated or laterally connected in COPI-SNARE RNAi-expressing photoreceptors (Figure 1B arrows).

### 3.2 Golgi stacks gathered in COPI-SNARE knockdown photoreceptors

**FIGURE 2:**
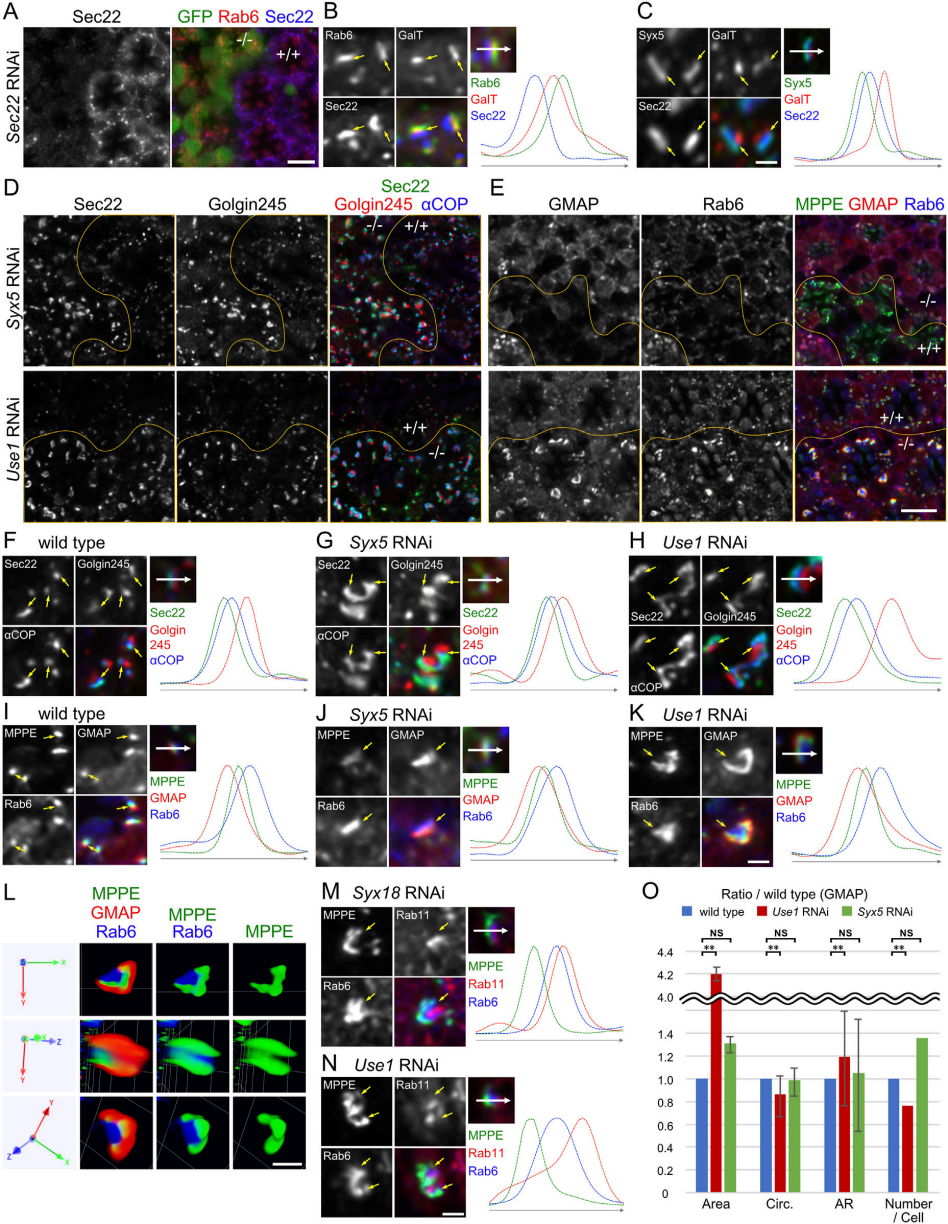
Golgi stacks are enlarged by knockdown of SNAREs for COPI fusion. **(A)** Immunostaining of Sec22 RNAi construct-expressing retina by eyeless-CoinFLP-Act5C-Gal4 using anti-Rab6 (red) and anti-Sec22 (blue) antibodies. GFP (green) represents the cells expressing Sec22 RNAi construct. **(B)** Left: wild-type Golgi stacks expressing GalT::CFP (red) immunostained by anti-Rab6 (green) and anti-Sec22 (blue) antibodies. Arrows indicate the relative position of staining. Right: the plot of signal intensities along the 1.5 μm from the top image. **(C)** Left: wild-type Golgi stacks expressing GalT::CFP (red) and Syx5::Myc immunostained by anti-Myc (green) and anti-Sec22 (blue) antibodies. Arrows indicate the relative position of staining. Right: the plot of signal intensities along the 1.5 μm from the top image. **(D)** Immunostaining of *Syx5* (upper) or *Use1* (lower) RNAi construct-expressing retina by eyeless-CoinFLP-longGMR-Gal4 (upper) or eyeless-CoinFLP-Act5C-Gal4 (lower) using anti-Sec22 (green), anti-Golgin245 (red) and anti-αCOPI (blue) antibodies. **(E)** Immunostaining of *Syx5* (upper) or *Use1* (lower) RNAi construct-expressing retina by eyeless-CoinFLP-longGMR-Gal4 (upper) or eyeless-CoinFLP-Act5C-Gal4 (lower) using anti-MPPE (green), anti-GMAP (red) and anti-Rab6 (blue) antibodies. **(F–H)** Left: wild-type **(F)**, *Syx5* knockdown **(G)** and *Use1* knockdown **(H)** Golgi stacks immunostained by anti-Sec22 (green), anti-Golgin245 (red) and anti-αCOPI (blue) antibodies. Arrows indicate the relative positions of staining. Right: the plot of signal intensities along the 1.5 μm from the top image. **(I–K)** Left: wild-type **(I)**, *Syx5* knockdown **(J)** and *Use1* knockdown **(K)** Golgi stacks immunostained with anti-MPPE (green), anti-GMAP (red), and anti-Rab6 (blue) antibodies. Arrows indicate the relative positions of staining. Right: the plot of signal intensities along the 1.5 μm from the top image. **(L)** Volumetrically rendered images of a *Use1* knockdown Golgi stack immunostained with anti-MPPE (green), anti-GMAP (red), and anti-Rab6 (blue) antibodies presented from three different angles. **(M, N)** Left: Golgi stacks with *Syx18* knockdown **(M)** and *Use1* knockdown **(N)** immunostained with anti-MPPE (green), anti-Rab11 (red), and anti-Rab6 (blue) antibodies. Arrows indicate the relative positions of staining. Right: the plot of signal intensities along the 1.5 μm from the top image.**(O)** Plots of the ratio of area, number, circularity (Circ), and aspect ratio (AR: major axis/minor axis) of Golgi stacks in *Use1* RNAi- or *Syx5* RNAi-expressing photoreceptors compared to those in wild-type photoreceptors. Blue, red, and green bars indicate wild-type, *Use1* RNAi, or *Syx5* RNAi-expressing photoreceptors, respectively. Error bars indicate standard SD of four retinas. Significance according to two-tailed unpaired Student’s t*-*test: **p < 0.01.Scale bars: 5 μm **(A,D, E)** and 1 μm **(B, C, F–K, L, M, N)**.

As shown in Figure 1B, which indicates changes in the shape and size of Golgi stacks in COPI-SNARE RNAi-expressing photoreceptors, we further investigated Golgi stack morphology in COPI- and COPII-SNARE RNAi-expressing photoreceptors. We examined the polarity of Golgi stacks using antibodies for Golgin245, αCOP, GMAP, MPPE, Rab6, and Sec22. Although the detailed localization of Golgin245, aCOP, GMAP, MPPE, and Rab6 has been previously demonstrated (Fujii et al., 2020a; Fujii et al., 2020b), the localization of Sec22 has not been investigated in fly retinas. Therefore, we first characterized an anti-Sec22 antibody. In Sec22 RNAi mosaic-expressing retina immunostained with anti-Sec22 and anti-Rab6 antibodies, dot-like staining of Sec22 was detected near Rab6 in wild-type photoreceptors (Figure 2A). However, this staining was absent in the photoreceptor expressing Sec22 RNAi construct (Figure 2A). Thus, both the anti-Sec22 antibody and Sec22 RNAi construct were functional. Sec22 was localized on the *cis*-side of CFP::GalT but on the slightly trans-side of Syx5::myc (Figures 2B,C). The polarity of Golgi stacks in *Syx5* RNAi-or *Use1* RNAi-expressing photoreceptors was maintained; the slight cis-localization of Sec22 against αCOP was not affected in *Syx5* RNAi-or *Use1* RNAi-expressing photoreceptors (Figures 2D,F–H). The order of localization of GMAP, MPPE, and Rab6 was also the same in wild-type, *Syx5* RNAi, and *Use1* RNAi-expressing photoreceptors (Figures 2E,I–K).

As shown in Figure 1B, in *Use1* RNAi-expressing photoreceptors, the Golgi apparatus appeared longer and larger than a single Golgi stack in wild-type cells (Figure 2D, E lower panels). This single large Golgi apparatus can be either a large single Golgi stack or a cluster of Golgi stacks. As shown in Figure 2K, MPPE-positive cisternae appeared to be separated into three distinct parts, suggesting that the large Golgi apparatus was a cluster of Golgi stacks. However, the Rab6-positive *trans*-Golgi networks (TGNs) appeared as a single large mass, and GMAP smoothly surrounded the MPPE-positive cisternae on the cis-side, indicating that the large Golgi apparatus was a single large Golgi stack. Figure 2L shows the large Golgi apparatus at three different angles. We have previously demonstrated that a similar cluster of Golgi stacks, which we named the BFA-Body, is formed upon administration of BFA (Brefeldin A) and that the center of the BFA-Body is composed of recycling endosomes (REs) (Fujii et al., 2020b). Investigation of the localization of the RE marker Rab11 revealed that RE is localized in the center of clustered Golgi stacks in *Syx18* or *Use1* RNAi-expressing photoreceptors (Figures 2M,N). Thus, the clustered Golgi stacks formed in *Use1* RNAi-expressing photoreceptors have a configuration similar to that of BFA-bodies, although the latter to contain a greater number of Golgi stacks.

We compared the area and number of Golgi stacks appearing in the optical sections of wild-type, *Use1* RNAi-expressing photoreceptors, or *Syx5* RNAi-expressing photoreceptors. Area per each continuous Golgi stack, which may reflect the fluorescence intensity rather than the actual area, was expanded using either *Use1* RNAi or *Syx5* RNAi. In contrast, the number of Golgi stacks decreased only by *Use1* RNAi. These results demonstrate that Golgi stacks are clustered by *Use1* RNAi but not by *Syx5* RNAi. To evaluate cisternal elongation in Golgi stacks, circularity (Circ) and the ratio of major to minor diameters when the Golgi stack is considered an ellipse (AR) were measured. The results showed that Circ was significantly decreased but AR was increased in *Use1* RNAi-expressing photoreceptors. In contrast, there is only little change of Circ and AR in *Syx5* RNAi-expressing photoreceptors. These results indicate that Golgi stacks are laterally expanded in *Use1* RNAi-expressing photoreceptors, but not in *Syx5* RNAi-expressing photoreceptors, compared to that in wild-type photoreceptors (Figure 2O).

### 3.3 Vesicle clusters and enlarged Golgi stacks in COPI- and COPII-SNARE knockdown photoreceptors

**FIGURE 3:**
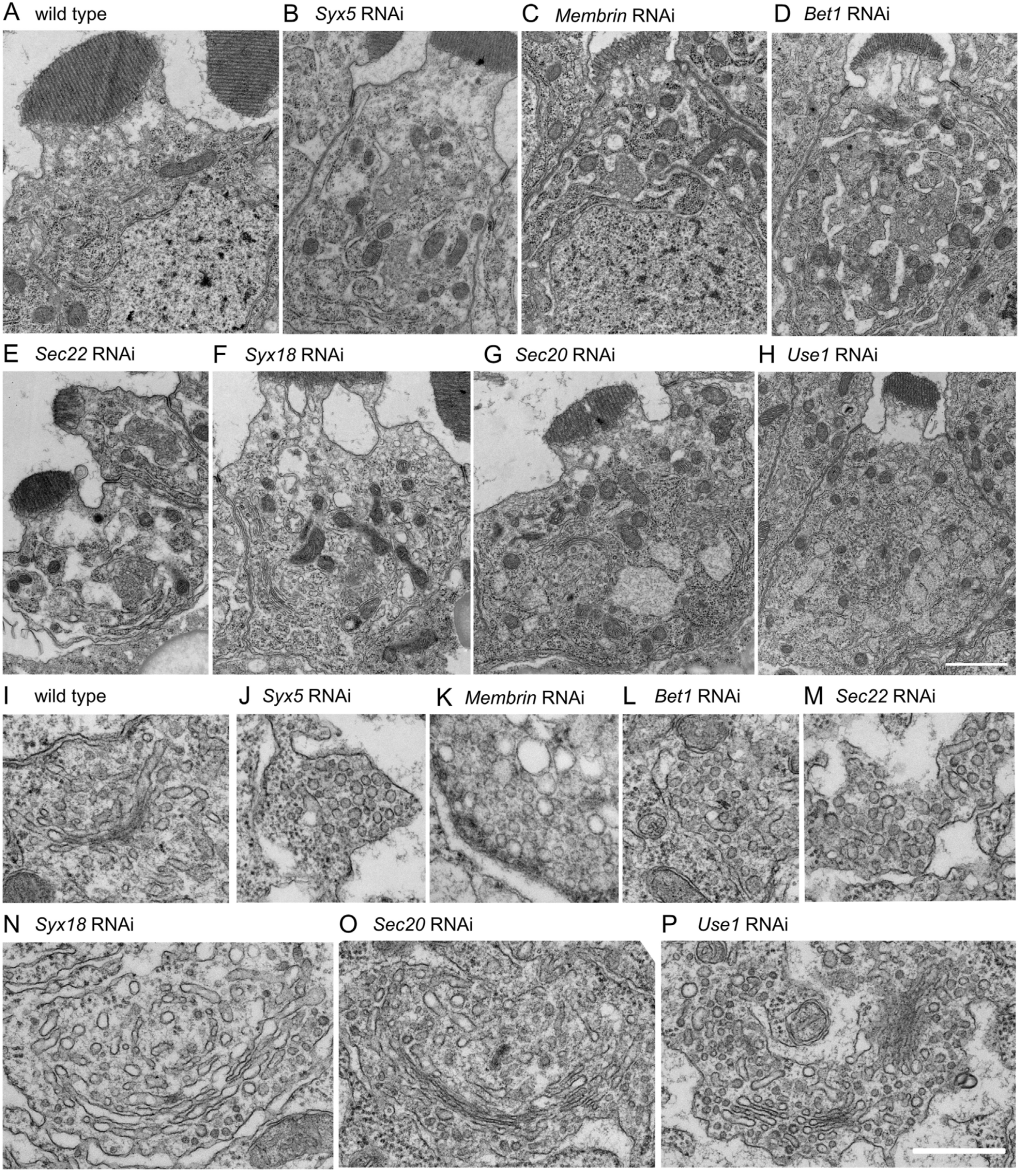
Morphologies of Golgi stacks in COPI- or COPII- SNARE knockdown photoreceptors. **(A–H)** Electron micrographs of SNARE RNAi construct-expressing photoreceptors by eyeless-CoinFLP-longGMR-Gal4 (*Syx5*, *Bet1*, *Syx18*, and *Sec20*) or eyeless-CoinFLP-Act5C-Gal4 (*Membrin*, *Sec22* and *Use1*) at late pupae. **(I–P)** Golgi stack or vesicle clusters in COPI- or COPII-SNARE knockdown photoreceptor. Scale bars: 2 μm **(A–H)** and 500 nm **(I–P)**.

To investigate how the structure of the Golgi stack was affected by COPI- and COPII-SNARE knockdown, we examined thin sections using electron microscopy (Figure 3). We found that there was no clear Golgi stack, but rather many vesicle clusters, in the COPII-SNARE knockdown photoreceptors (Figures 3B–E, J–M). We also observed that the ER membrane expanded, and the lumen was often dilated. These phenotypes resembled those observed in photoreceptors expressing Rab1 dominant-negative proteins (Satoh et al., 1997). In contrast, we observed enlarged Golgi stacks with long cisternae in COPI-SNARE knockdown photoreceptors (Figures 3F–H, N, O). Occasionally, Golgi stacks without lateral connections accumulated to form clustered Golgi stacks (Figure 3P). Both enlarged and clustered Golgi stacks were accompanied by numerous small vesicles. Additionally, we observed that the ER membrane was expanded, and the lumen was often dilated in all cases of COPI-SNARE knockdown, similar to COPII-SNARE knockdown.

**FIGURE 4:**
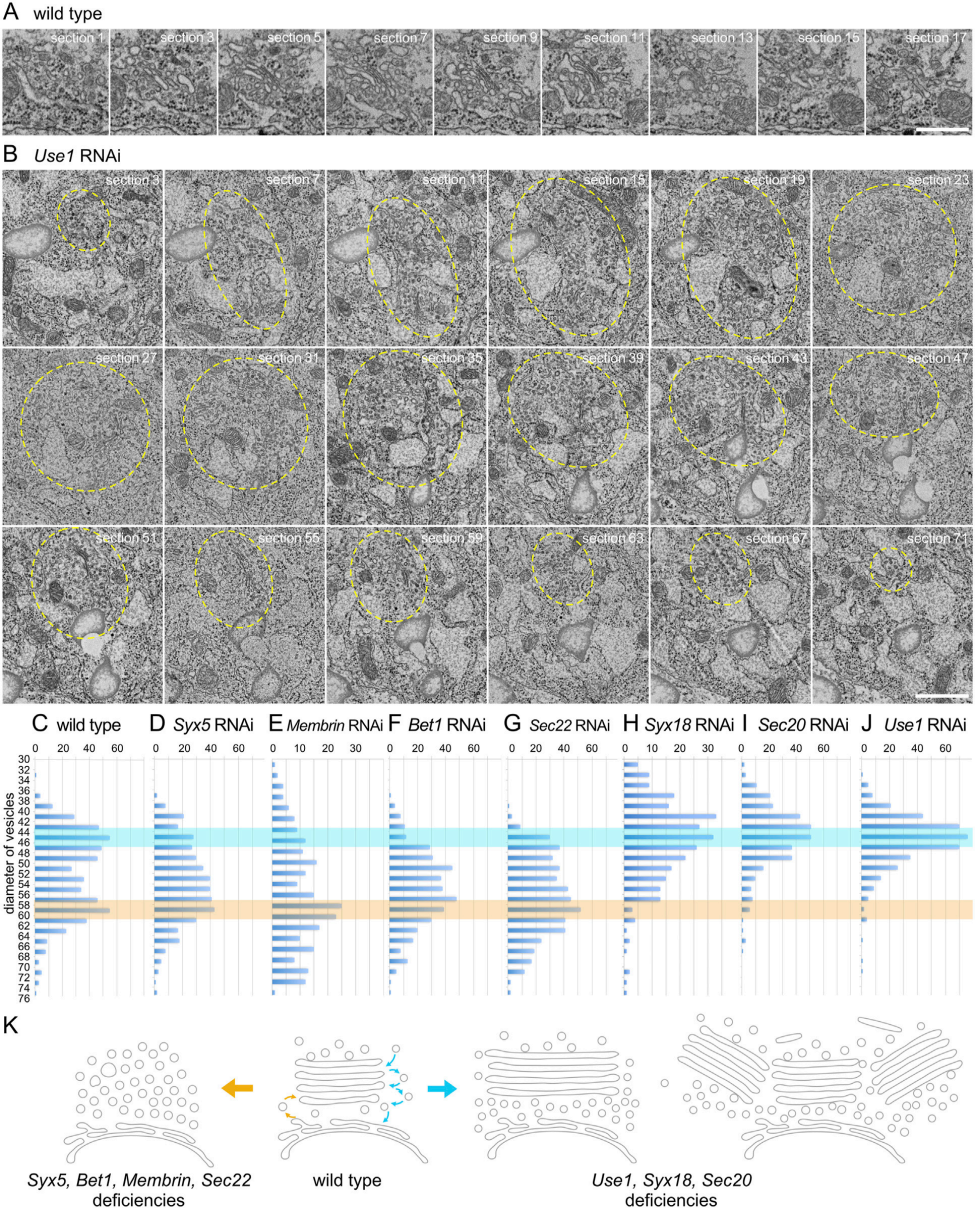
Enlarged Golgi stack in *Use1* knockdown photoreceptor. **(A)** Serial sections of a Golgi stack at 50-nm intervals in the wild-type photoreceptor, numbered as indicated. **(B)** Serial sections of a cluster of Golgi stacks at 50-nm intervals in the *Use1* RNAi construct expressing photoreceptor by eyeless-CoinFLP-longGMR-Gal4, numbered as indicated. A cluster of Golgi stacks circled with yellow line. **(C–J)** Plots of the number of vesicles with indicated diameters found near Golgi stacks or vesicle clusters in the wild-type **(C)** or SNARE RNAi construct-expressing photoreceptors by eyeless-CoinFLP-longGMR-Gal4 (*Syx5* **(D)**, *Bet1* **(F)**, *Syx18* **(I)**, and Sec2*0* **(J)** or eyeless-CoinFLP-Act5C-Gal4 (*Membrin* **(E)**, Sec2*2* **(G)** and *Use1* **(H)**) at late pupae. **(K)** Model of structural changes in Golgi stacks in COPI- and COPII-SNARE knockdown photoreceptor cells. COPII-SNARE knockdown transformed Golgi stacks into vesicle clusters with the same diameter as COPII vesicles (left). In contrast, with COPI-SNARE knockdown, Golgi stacks expanded or assembled around TGNs (right). These expanded or clustered Golgi stacks were accompanied by vesicles with COPI vesicle diameters. Scale bars: 500 nm **(A, B)**.

To gain a comprehensive understanding of enlarged and clustered Golgi stacks, we conducted serial sectional observations of a Golgi stack in a wild-type photoreceptor and clustered Golgi stacks in *Use1* RNAi-expressing photoreceptors. Although sections were prepared and observed at 50-nm intervals, the wild-type Golgi stack was presented at 100-nm intervals (Figure 4A; Supplementary Figure S1A) and a cluster of Golgi stacks in *Use1* knockdown was presented at 200-nm intervals (Figure 4B). Typical wild-type Golgi stacks in fly photoreceptors are less than 1 μm in length or depth. In contrast, enlarged Golgi stack/clustered Golgi stacks in *Use1* RNAi-expressing photoreceptors exceed 3 μm in diameter of the whole area. In *Use1* RNAi enlarged Golgi stack/clustered Golgi stacks, there was a long cisterna in section 17 and section 21, which exceeded 1 μm in length; however, most of cisternae were not connected and a couple of Golgi stacks simply gathered without clear connections. We also performed serial sectioning of vesicle clusters in *Bet1* RNAi-expressing photoreceptors (Supplementary Figure S1B). Again, although sections were prepared and observed at 50-nm intervals, they were presented at 300-nm intervals. Many discrete vesicle clusters, but not Golgi cisternae, were observed. The size of the vesicle clusters, typically less than 1 μm in length or depth, was comparable to Golgi stacks in the wild types. All sections with 50-nm intervals are presented as the supplemental movies (Supplementary Movie S1–S3).

### 3.4 Accumulation of COPI and COPII vesicles in COPI- and COPII-SNARE knockdown photoreceptors

Numerous vesicles were observed in both COPI- and COPII-SNARE knockdown photoreceptors, although their diameters differed. Therefore, we measured the diameter of vesicles near the Golgi stacks and counted the number of vesicles with each diameter. In wild-type Golgi stacks, we observed two peaks in the vesicle diameter distribution at approximately 42–48 nm and 56–62 nm (Figure 4C). The former corresponds to the diameter of COPI vesicles, whereas the latter corresponds to the diameter of COPII vesicles. In the COPII-SNARE knockdown Golgi stacks, the latter peak remained evident, whereas the former peak was difficult to discern (Figures 4D–G). Only Sec22 RNAi knockdown Golgi stacks exhibited a small peak in the 42–48 nm range (Figure 4G). Conversely, in COPI-SNARE knockdown Golgi stacks, the former peak of vesicle diameter around 42–48 nm was prominent, whereas the latter peak was absent (Figures 4H–J). These results indicated that COPI vesicles accumulated in COPI-SNARE knockdown Golgi stacks, whereas COPII vesicles accumulated in COPII-SNARE knockdown Golgi stacks, which perfectly matched our expectations.

## 4 Discussion

In this study, we examined the structural alterations of Golgi stacks in photoreceptor cells following the knockdown of COPI- or COPII-SNAREs. The results showed that when COPII-SNAREs were knocked down, Golgi stacks were transformed into clusters of vesicles with diameters similar to those of COPII vesicles. In contrast, when COPI-SNAREs were knocked down, Golgi stacks enlarged and often gathered in the TGN. These enlarged and clustered Golgi stacks were accompanied by vesicles with diameters similar to those of COPI vesicles. The results are schematically shown in Figure 4K.

Fragmentation of Golgi stacks and transformation into vesicle clusters have been previously described in the Sec22 mutant in plants (El-Kasmi et al., 2011), a hypomorphic mutant of *Syx5* in fly photoreceptors (Satoh et al., 2016), and the expression of dominant negative Rab1 in fly photoreceptors (Satoh et al., 1997). As Sec22, Syx5, and Rab1 are essential for the fusion of COPII vesicles with cis-Golgi cisternae, these vesicles in the vesicle clusters are supposed to be COPII vesicles which failed to fuse into the cisternae. Indeed, the diameters of the vesicles in the clusters formed in COPII-SNARE knockdown match well with those of the COPII vesicles. The loss of well grown cisternae under the COPII-SNARE knockdown can also be explained by the deficiency of COPII fusion which is required to form new cis-cisternae. Despite the loss of Golgi cisternae, the Golgi markers retained their polarized distribution. Vesicles in vesicle clusters may contain different sets of Golgi markers, depending on their *cis-trans* positioning.

We found that COPI-SNARE knockdown induced ER expansion, enlargement of Golgi stacks, and cluster formation of Golgi stacks in fly photoreceptors. ER expansion has been previously reported in *Use1*-deficient yeast (Belgareh-Touzé et al., 2003) and mammalian *Use1* KO cells (Uemura et al., 2009); however, to the best of our knowledge, enlargement or cluster formation of Golgi stacks in COPI-SNARE deficiency has not been reported. One reason for this may be the difficulty in assessing such a phenotype in mammalian cells or yeast due to the laterally conjugated Golgi ribbon in wild-type mammalian cells or unstacked Golgi cisternae in yeast.

Sec22 has been reported to regulate not only COPII vesicle fusion, but also COPI vesicle fusion in yeast and mammalian cells (Linders et al., 2019). While Sec22 knockdown in our study resulted primarily in COPII-SNARE deficient phenotypes including transformation of Golgi stacks into vesicle clusters rather than enlargement or clustering, Sec22 RNAi-treated cells also accumulated COPI and COPII vesicles, in contrast to other COPII-SNARE RNAi cells. The epistatic nature of COPII phenotypes to COPI phenotypes provides Sec22 knockdown cells with COPII phenotype. However, Sec22 must also be involved in COPI vesicle fusion.

We have previously reported that Golgi stacks in *Drosophila* S2 cells are highly mobile and undergo repetitive fusion and fission via TGNs. When ARFGEF Sec71 is impaired by BFA, Golgi stacks move constantly without affecting TGN-TGN fusion; however, TGN fission is suppressed. As a result, all Golgi stacks gather in the TGN to form a BFA body. The Golgi clusters found in the COPI-SNARE knockdown photoreceptors were quite similar to those in the BFA-bodies. Therefore, under COPI-SNARE knockdown, there must be either suppressed fission or enhanced fusion of TGNs. In addition to BFA bodies, enlarged Golgi stacks and Golgi clusters formed by COPI-SNARE knockdown resemble those of the cluster of Golgi stacks and RE around the centrosome, often found in HeLa or MDCK cells, and quite common in COS-1 cells (Misaki et al., 2010). A recent report indicated that the Golgi complex in mammalian cells is assembled by a number of “Golgi units” with a diameter of 1–3 μm, and that these Golgi units undergo dynamic separation and fusion under normal and nocadazole-treated or removed conditions (Harada et al., 2024). This similarity is consistent with the idea that the variety in the cell-wide appearance of the Golgi system reflects the difference in the kinetic balance between fission and fusion of TGNs or Golgi units.

## Data Availability

The original contributions presented in the study are included in the article/Supplementary Material, further inquiries can be directed to the corresponding authors.
