# Supplementary material for: Golgi clustering by the deficiency of COPI-SNARE in Drosophila photoreceptors
Source: Front Cell Dev Biol. 2024 Sep 4;12:1442198. doi: 10.3389/fcell.2024.1442198 (PMC11408282; doi:10.3389/fcell.2024.1442198)
Supplement: Supplementary file 3 [file DataSheet1.pdf]

Supplementary Material

Tago et al., Figure S1

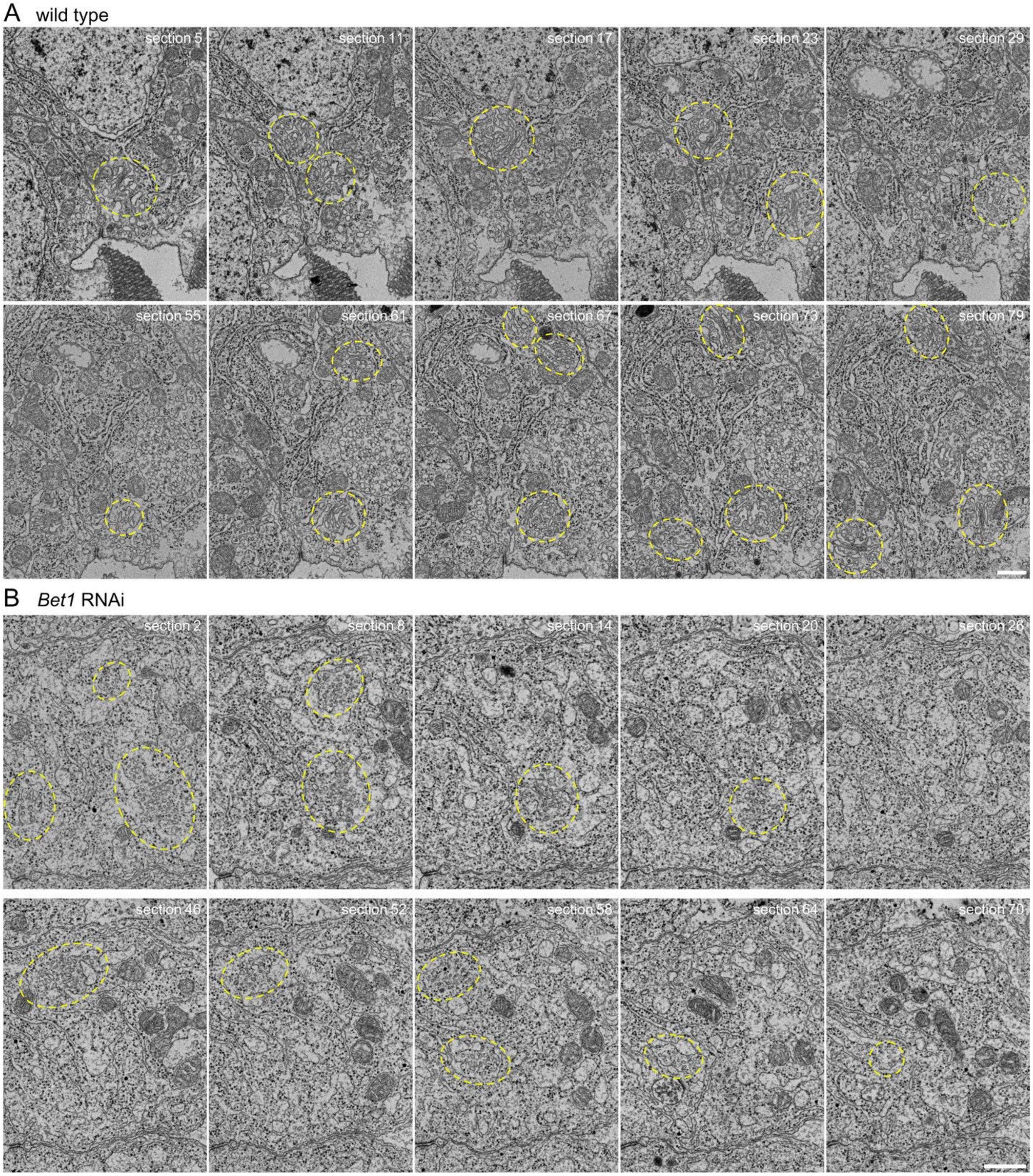

Supplementary Figure 1. Vesicle clusters in Syx5 knockdown photoreceptor.

(**A**) Serial sections of Golgi stacks at 50-nm intervals in the wild-type photoreceptor, numbered as indicated. Golgi stacks circled with yellow lines.

(**B**) Serial sections of Vesicle clusters at 50-nm intervals in the Bet1RNAi construct-expressing photoreceptors using eyeless-CoinFLP-lonGMR-Gal4, numbered as indicated. Vesicle clusters circled with yellow lines.

Scale bars: 500 nm (A, B).

Movie 1. Golgi stacks in wild-type photoreceptors

Serial sections of Golgi stacks at 50-nm intervals in the wild-type photoreceptor.

Scale bars: 500 nm.

Movie 2. Golgi stacks in Use1 knockdown photoreceptors

Serial sections of Golgi stacks at 50-nm intervals in the Use1RNAi construct-expressing photoreceptors using eyeless-CoinFLP-Act5C-Gal4.

Scale bars: 500 nm.

Movie 3. Golgi stacks in Bet1 knockdown photoreceptors

Serial sections of Golgi stacks at 50-nm intervals in the Bet1RNAi construct-expressing photoreceptors using eyeless-CoinFLP-longGMR-Gal4.

Scale bars: 500 nm.
